# When Guidelines Collide: Individualized Care Across Overlapping Cerebrovascular Emergencies

**DOI:** 10.1016/j.jaccas.2026.110241

**Published:** 2026-10-14

**Authors:** Alhamza R. Al-Bayati

**Affiliations:** UPMC Stroke Institute, University of Pittsburgh, School of Medicine, Pittsburgh, Pennsylvania, USA

**Keywords:** anticoagulation, bubble echocardiography, computed tomography, hemorrhage, pulmonary circulation, thrombosis

**Figure:**
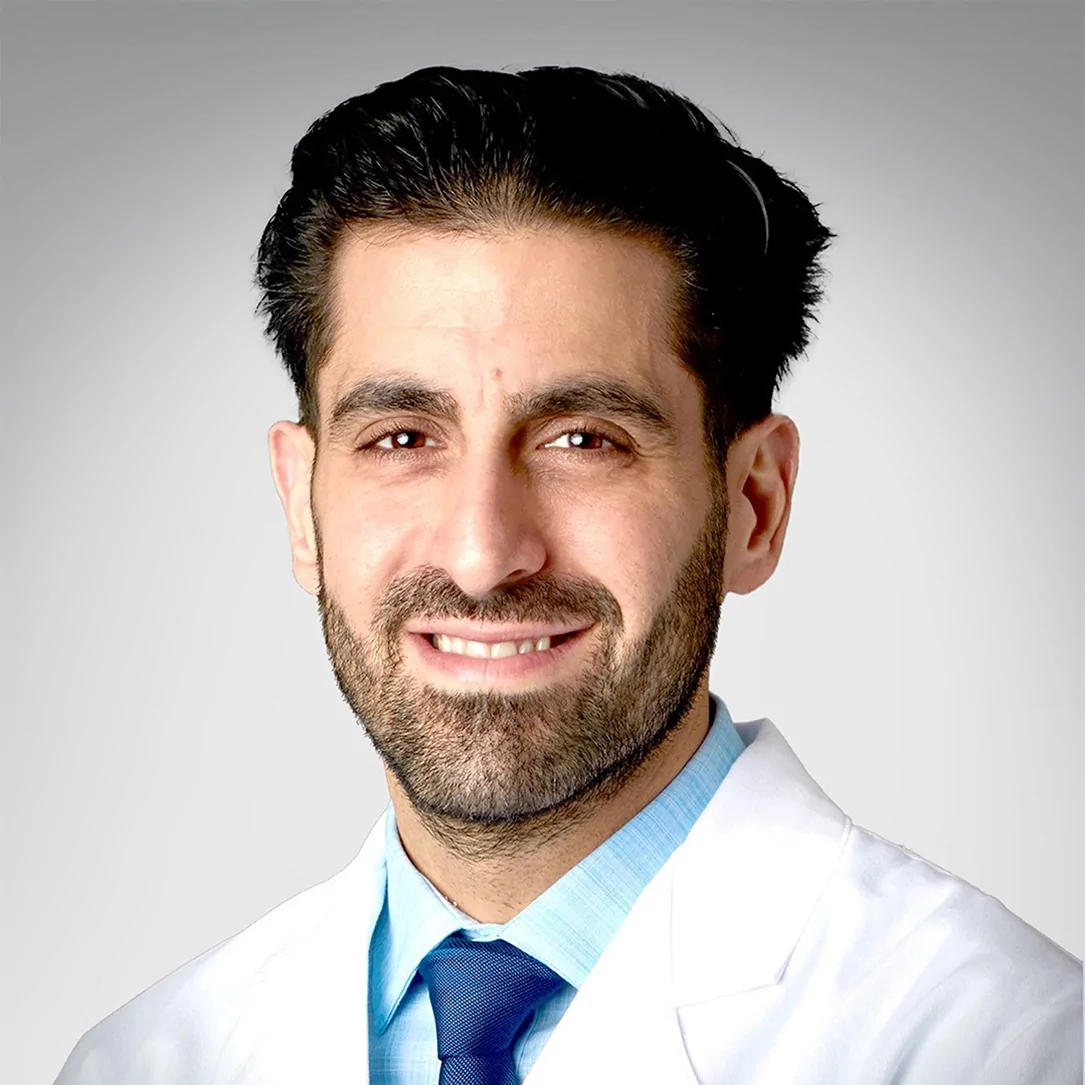

The case report by Luna-Alcala et al in this issue describes a 74-year-old woman whose hospitalization moved through nearly every high-stakes decision point in contemporary cerebrovascular medicine: wake-up stroke thrombolysis, hemorrhagic transformation, intermediate-risk pulmonary embolism, recurrent large-vessel occlusion, and paradoxical embolism confirmed by direct visualization of thrombus straddling a patent foramen ovale.^1^ Read in isolation, each of these problems has a guideline. Read together, as this patient experienced them, the guidelines argue with each other. That tension, more than any single intervention described, is the real subject of this report, and it deserves comment.

Reperfusion selection in this case relied on computed tomography (CT) perfusion rather than magnetic resonance imaging diffusion-weighted imaging fluid-attenuated inversion recovery MRI DWI-FLAIR mismatch, a departure driven by claustrophobia and the need for timely decision-making in a wake-up presentation. The trial evidence most clinicians reach for, WAKE-UP and EXTEND, was built on advanced imaging selection, MRI DWI-FLAIR mismatch in WAKE-UP and automated CT or MR perfusion mismatch in EXTEND.^2^^,^^3^ Since those trials, however, the evidence base for perfusion-guided thrombolysis has grown considerably. TRACE-III demonstrated benefit from tenecteplase given 4.5-24 hours after onset using CT or MRI perfusion selection in patients without access to thrombectomy, and CHABLIS-T II extended similar findings to a broader perfusion-selected study group.^4^^,^^5^ Taken together, this body of work supports CT perfusion as a legitimate, increasingly mainstream alternative to MRI-based selection, rather than a compromise used only when MRI is unavailable. What none of these trials fully addresses, and what this case illustrates well, is that CT perfusion characterizes hemodynamics far more reliably than it characterizes hemorrhage. This patient was later found to have both focal subarachnoid hemorrhage and hemorrhagic transformation that were not evident on the pretreatment scan. The report is candid about this limitation rather than glossing over it, and that candor is itself instructive. As perfusion-based selection spreads into centers without ready MRI access, this case is a useful reminder that the imaging surrogate carries its own blind spots, and that clinicians should document explicitly what was and was not excluded before treatment.

The pivotal decision in this case was full-dose anticoagulation for pulmonary embolism in a brain that had just bled. Stroke guidance counsels delay after hemorrhagic transformation, whereas pulmonary embolism guidance counsels prompt therapeutic anticoagulation to prevent hemodynamic collapse.^6^^,^^7^ Neither guideline was written with the other in the room. Real-world data help calibrate how much delay is typical, and how much may be too much. In a dual-center cohort of patients with intracranial hemorrhage after thrombolysis or thrombectomy, the median delay to any antithrombotic therapy was 24 hours after no hemorrhage, 26 hours after minor hemorrhage, and 39 hours after major hemorrhage, with a nonsignificant trend toward higher recurrence in the major-hemorrhage group.^8^ Emerging risk-adapted anticoagulation protocols for patients with atrial fibrillation and mild hemorrhagic transformation are similarly moving away from fixed windows toward individualized, imaging-guided restart criteria. Indeed, the 2026 American Heart Association/American Stroke Association acute ischemic stroke guideline now concludes that early direct oral anticoagulant initiation is safe in selected patients and that antithrombotic therapy can reasonably be initiated or continued even after hemorrhagic transformation, subject to individualized risk assessment.^9^ What the authors describe here—close monitoring, multidisciplinary input, and a willingness to reassess as new information arrived—sits squarely within that emerging paradigm rather than outside it. This is the part of the manuscript most worth teaching from, and future case series in this space would benefit from reporting these bedside deliberations in comparable detail, including the specific imaging or clinical triggers that would have prompted a different choice, ideally organized around the kind of consolidated timeline the authors provide.

The decision to proceed with mechanical thrombectomy for recurrent large-vessel occlusion, so soon after thrombolysis and hemorrhagic transformation, will strike some readers as aggressive. It is worth noting that repeated endovascular therapy for recurrent occlusion has been shown to be feasible and generally safe in a multicenter experience, even outside the setting of recent hemorrhage.^10^ That precedent supports the authors' choice here, though a single favorable outcome in 1 patient still cannot establish general safety, a point the take-home messages appropriately reflect. The broader lesson is that recurrent ischemia in a rapidly deteriorating patient can outweigh theoretical bleeding risk, and that this judgment belongs to the treating team at the bedside rather than to a static contraindication list.

Perhaps the most durable teaching point in this report concerns risk scoring itself, and the manuscript's discussion of the risk of paradoxical embolism (RoPE) score deserves to be pushed 1 step further. A RoPE score of 3 would have labeled this patient's paradoxical embolism risk as low, largely because RoPE weights clinical variables such as age and vascular risk factors and does not incorporate the anatomy of the interatrial septum. The 2024 European Stroke Organization guideline on patent foramen ovale (PFO) after stroke now recommends layering RoPE with the PFO associated stroke casual likelihood (PASCAL) classification, which explicitly adds shunt size and the presence of an atrial septal aneurysm to the causal assessment.^11^ This patient had both a 15 mm septal excursion and a large right-to-left shunt with more than 30 microbubbles crossing into the left atrium, features that under PASCAL would move her assessment toward a probable, rather than unlikely, PFO-associated mechanism well before the thrombus itself was ever visualized. Framed this way, the low RoPE score was not wrong; it was simply incomplete, and the transesophageal echocardiogram did not so much contradict the score as fill in a picture that the score was never designed to capture on its own. Clinicians reaching for RoPE in isolation, especially in patients with concurrent venous thromboembolism, should pair it with an anatomical classification before concluding that paradoxical embolism is unlikely.

Cases like this 1 argue for 2 things going forward. First, dynamic, updatable risk-assessment frameworks that explicitly accommodate competing thrombotic and hemorrhagic processes, rather than sequential application of disease-specific algorithms that assume the patient has only 1 problem at a time. Second, registry infrastructure capable of capturing these multisystem overlap presentations at scale, because no single case, however well-documented, can generate the evidence base that individualized decision-making ultimately needs. Efforts already underway within stroke and neurointerventional registries to track complex, overlapping presentations, and to document the shared decision-making that now governs PFO management in borderline cases, will be essential to turning bedside judgment calls like this one into generalizable guidance. Until then, well-documented cases such as this remain the field's best available teacher.

## Funding Support and Author Disclosures

Dr Al-Bayati has consulting and advisory roles with Johnson & Johnson MedTech, Stryker Neurovascular, Scientia Vascular, Balt, Imperative Care, and Anaconda Biomed; and holds stock options in Piraeus Medical and Hyperion Surgical.
